# Improving Microcirculation With Nerve Decompression: The Missing Link in Treatment of Diabetic Neuropathy and Diabetic Foot Ulcer

**DOI:** 10.1111/iwj.70198

**Published:** 2025-04-15

**Authors:** David Scott Nickerson, Dwayne Sonny Yamasaki

**Affiliations:** ^1^ NE Wyoming Wound Care Clinic Sheridan Wyoming USA; ^2^ Enso Medical Technologies, Inc. Saint Augustine Florida USA

**Keywords:** amputation in diabetes, autonomic neuropathy, entrapment neuropathy, skin microcirculation, surgery

## Abstract

Sympathetic dysfunction in skin is well known in diabetic peripheral neuropathy. This produces dry, cracked, peeling skin susceptible to infection and also epidermal microcirculation insufficiency. Impaired autonomic neurovascular control opens dermal arterio‐venous anastomoses and shunts microcirculation away from the epidermis and impairs skin oxygenation and nutrition. Few recognise that diabetic neuropathy includes swelling‐induced entrapment neuropathy. Multiple peripheral nerves, swollen by the secondary polyol metabolic pathway, suffer local compressions at fibro‐osseous tunnels. This includes the C‐fibres controlling autonomic functions which constitute most of the nerve axons. No current standard of care therapy addresses the sympathetic‐regulated neurovascular impairment of skin microcirculation in diabetes. Epineurolysis surgery for peripheral nerve decompression relieves local axonal compressions and generates recovery of sub‐epidermal capillary flow. Clinical and animal diabetes studies have demonstrated objective improvements to epidermal hypoxia, demyelination and axonal histology. Seven surgery studies find an average 1.39% recurrence and zero amputations after prior Risk Class 3 wound healing in a mean of 1.78 years of follow‐up. Deficits of electrophysiology, transcutaneous oxygenation and vasa nervorum circulation also improve. Surgically improved microcirculation is physiology‐based. Nerve decompression minimises diabetic peripheral neuropathy, avoids initial diabetic foot ulcers, promotes neuropathic diabetic foot ulcer healing and minimises ulcer recurrences and subsequent amputation. The observational studies of these important benefits suggest wide application to the complications of diabetes neuropathy and beg for academic attention to Level 1 EBM confirmation.

**Summary**

- Standard of care (SOC) methods for diabetic foot ulcer (DFU) treatment are beset bydelayed or failed healing, high recurrence rates, amputations, and early mortality.
- SOC offers no interventions that address epidermal microcirculation deficit and hypoxia.
- Observational studies of nerve decompression (ND) and neuropathic DFU (nDFU)recurrence show very large risk reductions from the 20%–40%/year of SOC meta‐analyses to < 2% annually in 7 ND reports.
- Impairment of autonomically regulated skin microcirculation in diabetes and DPN has been recognized for decades.
- A hyperactive polyol metabolic pathway in diabetes produces secondary peripheral nerve enlargement, local fibro‐osseous tunnel nerve compressions, and damaged autonomic andsensorimotor functions.
- Clinical and laboratory evidence shows surgical ND relieves entrapment neuropathy, improves both sensorimotor and autonomic nerve function, rejuvenates skin microcirculation and minimizes DFU recurrence risk.
- We elucidate the evidence for the points above explaining how ND surgery can achieve such robust benefit.
- ND is a physiologically based intervention in DPN which should attract Level 1 EBM research protocols to test whether or not this first treatment of skin microcirculation is as effective as observational studies suggest.

## 1 Introduction

The physiologic final common pathway of diabetic foot ulcers (DFU) is accepted to be skin hypoxia and ischaemia [1, 2]. We review in this article the literature connecting the secondary metabolic polyol pathway in diabetic hyperglycaemia to peripheral nerve entrapment neuropathy (EN), autonomic dysfunction, compromised microvascular circulation and epidermal hypoxia. This link has been largely overlooked in the literature. Several processes that can contribute to skin ischaemia are acknowledged to be present in feet with diabetic peripheral neuropathy (DPN) and widely considered to be causative of DFU. These factors include local skin pressure concentration [3], plantar callus formation, plantar fat pad atrophy and displacement [4], foot deformity, somatic sensory and motor neuropathy [5], macrocirculatory occlusive peripheral artery disease (PAD) [6] and altered cutaneous microcirculation [7]. Failure of capillary skin circulation is the terminal cause of epidermal hypoxia and ischemic tissue loss in the diabetic foot. We suggest relief of this EN explains how surgical nerve decompression (ND) reduces initial and recurrent risk of neuropathic DFU (nDFU) and other DPN foot complications.

## 2 Background

The motivation for a review of ND surgery in the diabetic foot was a 2019 recommendation by the International Working Group on the Diabetic Foot (IWGDF) advising, ‘for an active or imminent ulcer … we suggest not to use a nerve decompression procedure’. This was contrary to our clinical experience with DPN and understanding of DFU pathophysiology. Our aim, then, was to compare published studies using ND for DFU to the large literature reporting results of SOC.

Comparing ND and prior standard of care (SOC) in the DFU situation is a bit complicated. Recommended criteria for ND in DSPN candidates include adequate circulation [8]. So, ND use in DFU has been applied almost entirely to the neuropathic (nDFU) but not the neuroischemic situation. Conversely, the large literature of DFU SOC treatment and outcomes combines both nDFU and neuroischemic ulcers (n‐iDFU) in varying proportions which have changed over time.

For our analysis, a literature search was conducted from 1 June 2022 to 1 June 2024, and the following databases were screened: PubMed, Cochrane Library and Google Scholar. For clinical trials: Clinicaltrials.gov, Cochrane Central Register of Controlled Trials were screened. The following terms were used in various combinations: ‘diabetic foot’, ‘diabetic neuropathy’, ‘compression neuropathy’, ‘entrapment neuropathy’, ‘nerve decompression’, ‘external neurolysis’, ‘incidence’, ‘prevalence’, ‘meta‐analysis’, ‘systematic review’, ‘ulcer recurrence’, ‘clinical trial*’, ‘Eurodiale’, ‘standard of care’, ‘SOC’, ‘IWGDF’ and ‘International Working Group on the Diabetic Foot’. Studies were removed that were older than 20 years, SOC studies with fewer than 500 subjects, or used the following terms: ‘quality of Life’, ‘QOL’, ‘carpal tunnel’, ‘CTS’, ‘ulnar tunnel’, ‘guidelines’ and ‘protocol’. Based on our screening of the literature, the seven ND studies cited here are the only published studies that include data on DFU recurrence and post‐healing amputation.

### 2.1 Micro‐Circulation in Diabetes

**FIGURE 1:**
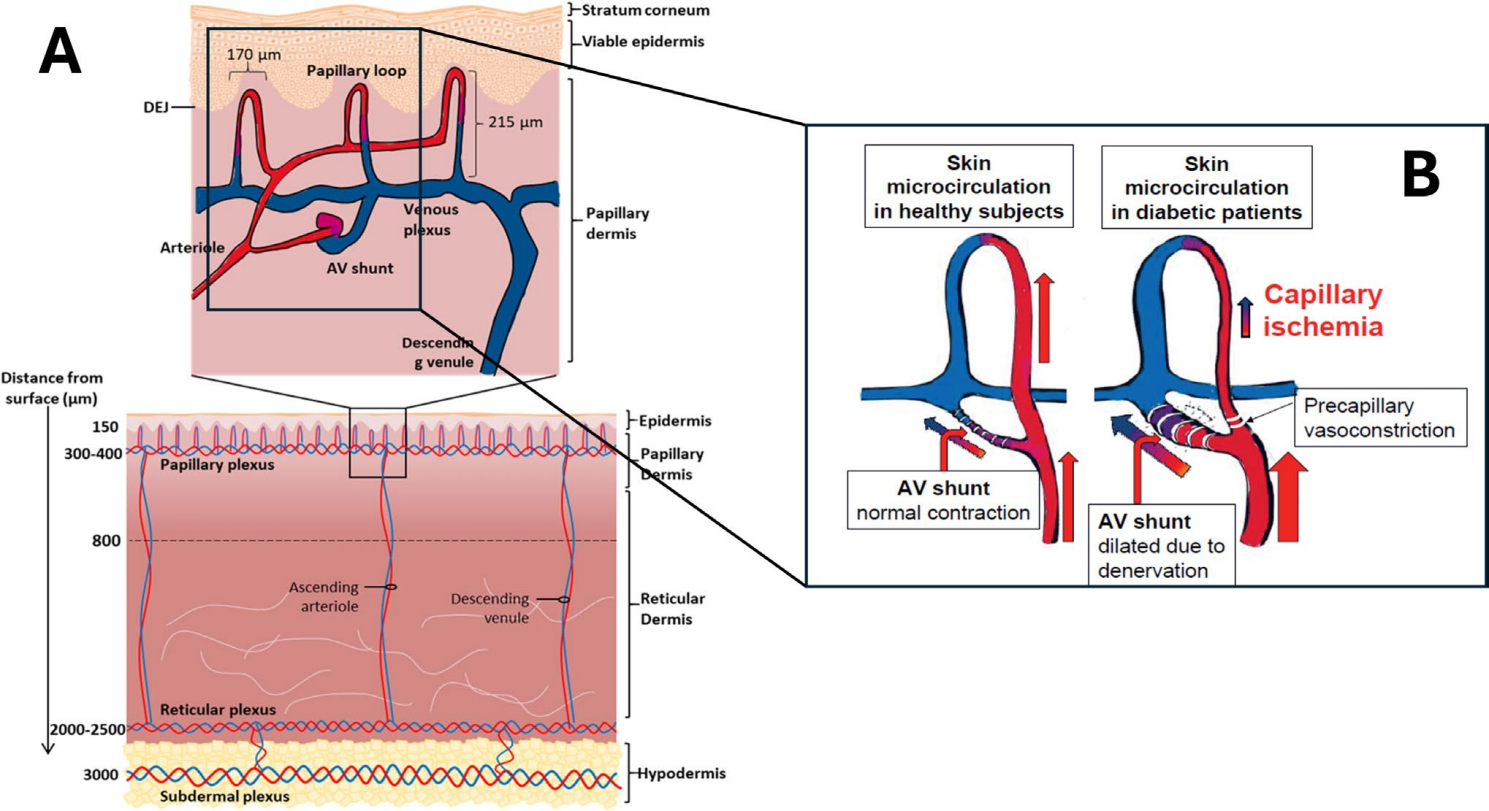
(A) Illustration of the epidermal and dermal circulation; (B) The altered blood flow pattern in diabetes. With sympathetic paralysis, Increased flow through the a‐v anastomosis shunts blood directly into the venous outflow. Combined with a pre‐capillary constriction, this robs the epidermis of oxygenation and nutrition [7]. With permission from Lectorio (A), and Prof. Bengt Fagrell and Prof. Gun Jorneskog (B).

Watkins and Edmonds four decades ago described increased pedal blood flow and venous oxygenation, and identified arterio‐venous (a‐v) shunting as significant in DPN and the diabetic neuroarthropathy of Charcot foot [9]. Greenman et al. specifically identified epidermal hypoxia, consistent with a‐v shunting, in the skin of patients with diabetes. They observed this impairment to be accentuated in the presence of neuropathy [10]. The a‐v anastomoses in the dermis, under sympathetic control, can divert blood away from the sub‐epidermal capillaries and directly into the venous outflow. In the normal microcirculatory state, sympathetic activity closes the a‐v anastomoses, preventing shunting and modulates suitable blood flow to the epidermis. DPN abolishes that autonomic control, allowing shunting to produce an a‐v vascular steal. Simultaneously, precapillary vasoconstriction also occurs, upstream of the epidermal subpapillary capillaries. These events restrict both the flow into, and pressure drop across, the sub‐epidermal capillary plexus (Figure 1). That condition of restricted epidermal blood supply in diabetes is described as chronic capillary ischaemia (CCI) [7, 11]. Microcirculation flow is found to be impaired even more if DPN and DFU are both present [12, 13].

**FIGURE 2:**
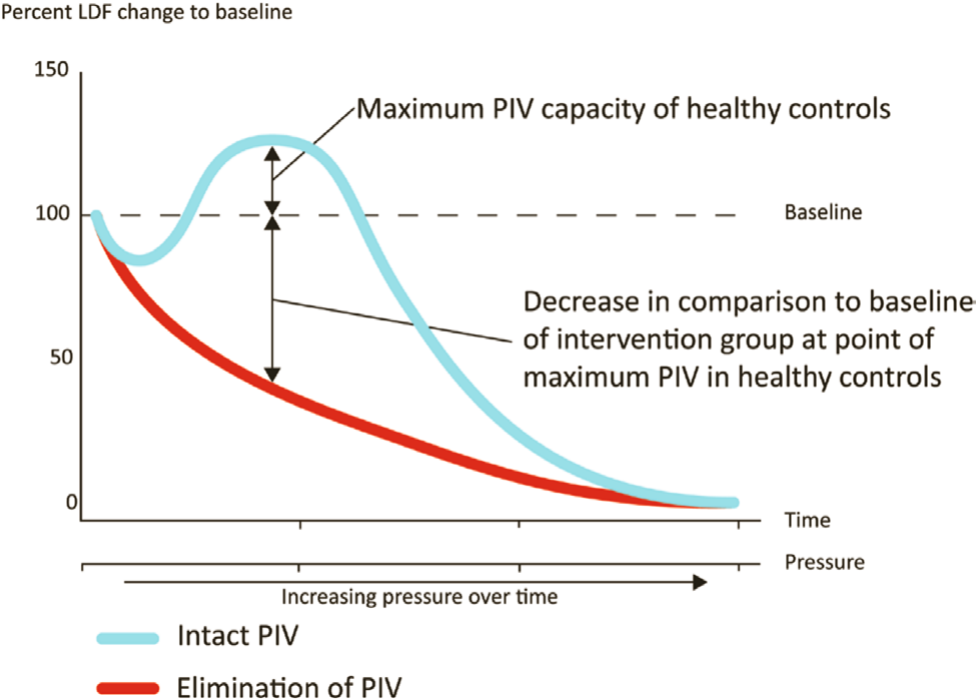
Quantitative example showing deficient epidermal blood flow (red) in diabetic neuropathy subjects in comparison with normal pressure‐induced vasodilation (blue). With permission from Zwanenberg [14].

Another little‐known neurocirculatory mechanism present in diabetes is ‘pressure‐induced vasodilation’ (PIV). The skin has mechanoreceptors which generate afferent pressure information that can actuate an efferent skin vasodilatation response. When moderate pressure is gradually applied, skin oxygenation as measured by transcutaneous oximetry (tcpO2) typically increases about 45%, as Figure 2 shows [14, 15]. A pronounced deficit of PIV in response to skin pressure accretion and concentration can generate a local situation of relative skin ischaemia and nutritional deficit. This increases the diabetic foot's vulnerability to epidermal and dermal necrosis, DFU development and impaired healing at the pressure site [16]. PIV is found in mice, rats and humans. This protective autonomic reflex is diminished in diabetes, more severely so in DFU [17], and is improved in animal diabetic states by ND surgery [18, 19, 20].

Other measures of vascular function are also improved by ND, including pulsatility index, distal arterial flow volumes and peripheral resistance [21]. Posterior tibial artery flow increases > 60% after tarsal tunnel ND in hard‐to‐heal nDFU wounds (Pejkova, personal communication).

Peripheral nerve vasa nervorum circulation is decreased in diabetic states but improved by ND [22]. Uemura et al., using SPY indocyanine green fluorescence, described > 30% local perineural blood flow improvement during endoscopic ND of the diabetic tibial nerves of tarsal tunnel syndrome [23]. Jung et al. describe improved vascular flow in connection with ND after chronic nerve compressions [24]. The acknowledged benefit of SOC treatments incorporating plantar pressure offloading presumably derives from reducing mechanical compression of capillaries occurring with ambulation and standing. Flow‐reducing compression effects and the ongoing presence of EN‐based CCI and PIV dysfunction would combine in producing skin hypoxia. With ND, rejuvenation of autonomic function with improved neural vascular flow and epidermal microcirculation revival would provide additional benefit to the SOC plantar unloading which presently is the gold standard pathway to best DFU healing rates.

### 2.2 Entrapment Neuropathy and ND in Diabetes Literature

The sensorimotor impairment of DPN is classically described as length dependent axonopathy (LDA) and is thought to be the primary factor permissive of DFU. The LDA is recognised as being progressive, resistant to pharmacology and irreversible once established [1, 25, 26]. A recent *Lancet Neurology* review discusses DPN aetiology and DFU pathogenesis, conceding that the LDA process is complex and understanding is incomplete [27]. It primarily focuses on the metabolic syndrome as a critical risk factor. Proposed mechanisms converge on a unifying theme of bioenergetic failure in the peripheral nerves due to their unique anatomy. There is no mention of nerve enlargement, EN or neurovascular effect. Much academic thought on DPN and DFU aetiology similarly ignores compression neuropathy. An in‐depth summary of skin innervation and impaired wound healing in diabetes by Nowak et al. describes the DPN state only as ‘length dependent dying back of axons’ [28]. The ADA Standards of Care in Diabetes—2023 makes no mention of EN or altered autonomic control of epidermal microcirculation in DPN [29]. Likewise, a current JAMA review of DFU fails to mention EN or microcirculation deficit [30]. Calcutt et al. note existence of the polyol pathway but without acknowledgement of the osmotically driven nerve enlargement and ensuing compression effects [31]. Javed et al. review painful DPN without considering nerve enlargement and entrapment [32], as do Feldman et al. [33] Although many diabetologists do recognise autonomic neuropathy, it is usually in relation only to dry skin, reduced sweating, orthostatic intolerance or cardiac, GI or GU dysfunction as Wraibel et al. assert in their 2023 *Frontiers in endocrinology* literature review [2, 28, 34].

## 3 Review of Nerve Decompression for DPN

### 3.1 Awareness of Entrapment Neuropathy and Nerve Compression

**FIGURE 3:**
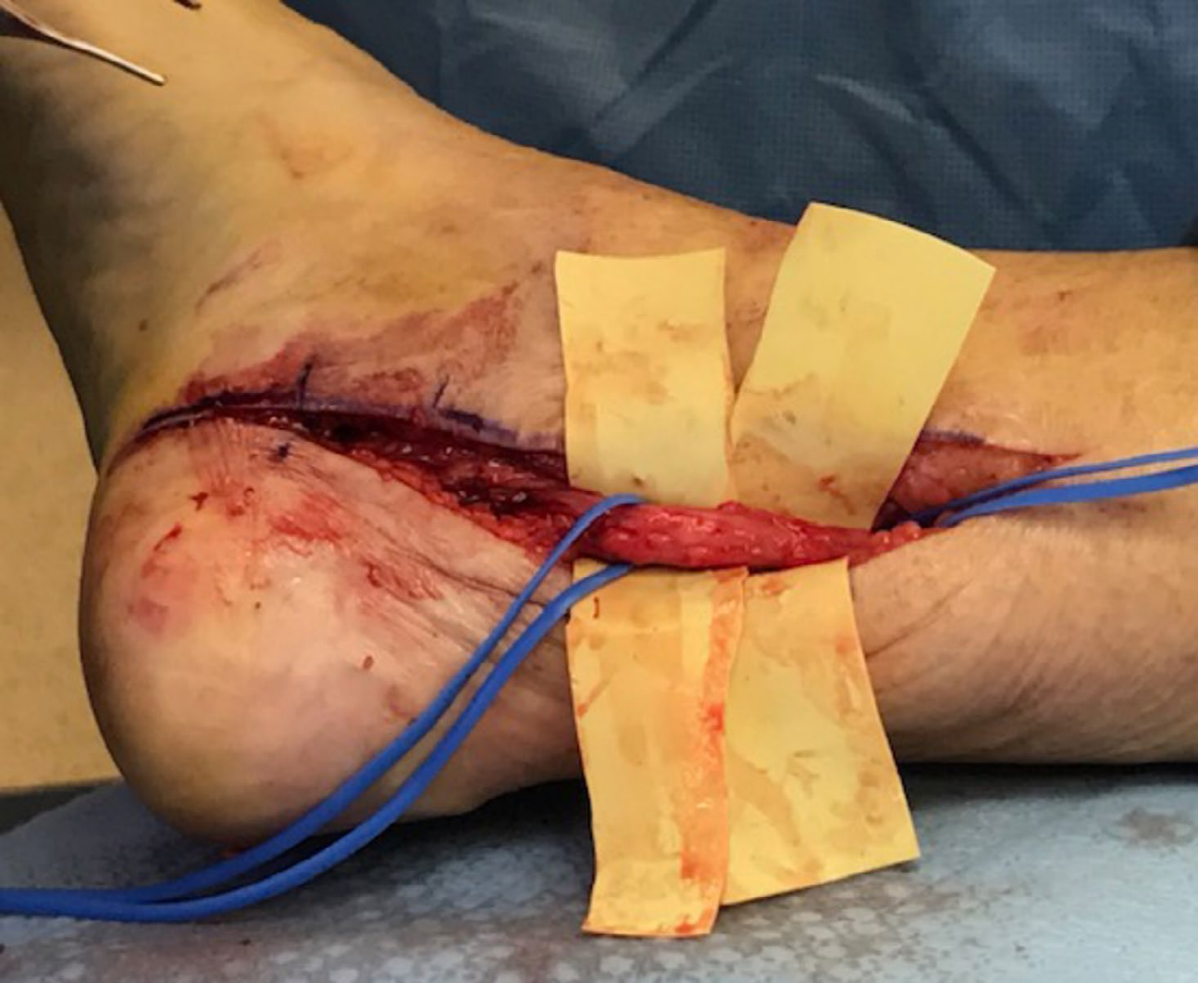
An operative photograph after release of the medial retinaculum at the tibial malleolus in a diabetic patient demonstrates the enlargement and edematous appearance of the posterior tibial nerve where it separates into medial calcaneal, medial plantar and lateral plantar nerves. Courtesy of Ryan Pereira, DPM.

The swollen, enlarged nerves in diabetes have been demonstrated multiple times with ultrasound or MRI imaging [35, 36, 37]. In general, little attention has been given to the fact that multiple peripheral nerve enlargements, the subsequent nerve trunk compressions in fibro‐osseous tunnels, and EN are common secondary effects of the diabetes metabolic abnormalities [36, 38, 39]. A general unfamiliarity with EN is perhaps understandable. Medical practitioners, endocrinologists and neurologists treating DPN and DFU patients never see for themselves the swollen, pale, watery diabetic peripheral nerves as surgeons do. Hence, they may not be cognizant of this common pathology and the extent of its severity, as Figure 3 shows. Still, neurologists Rota and Morelli find ENs to be so frequent at any stage of diabetic disease that in their experience, ‘they may be considered a neurophysiological hallmark of peripheral nerve involvement in diabetes mellitus' [38].

**FIGURE 4:**
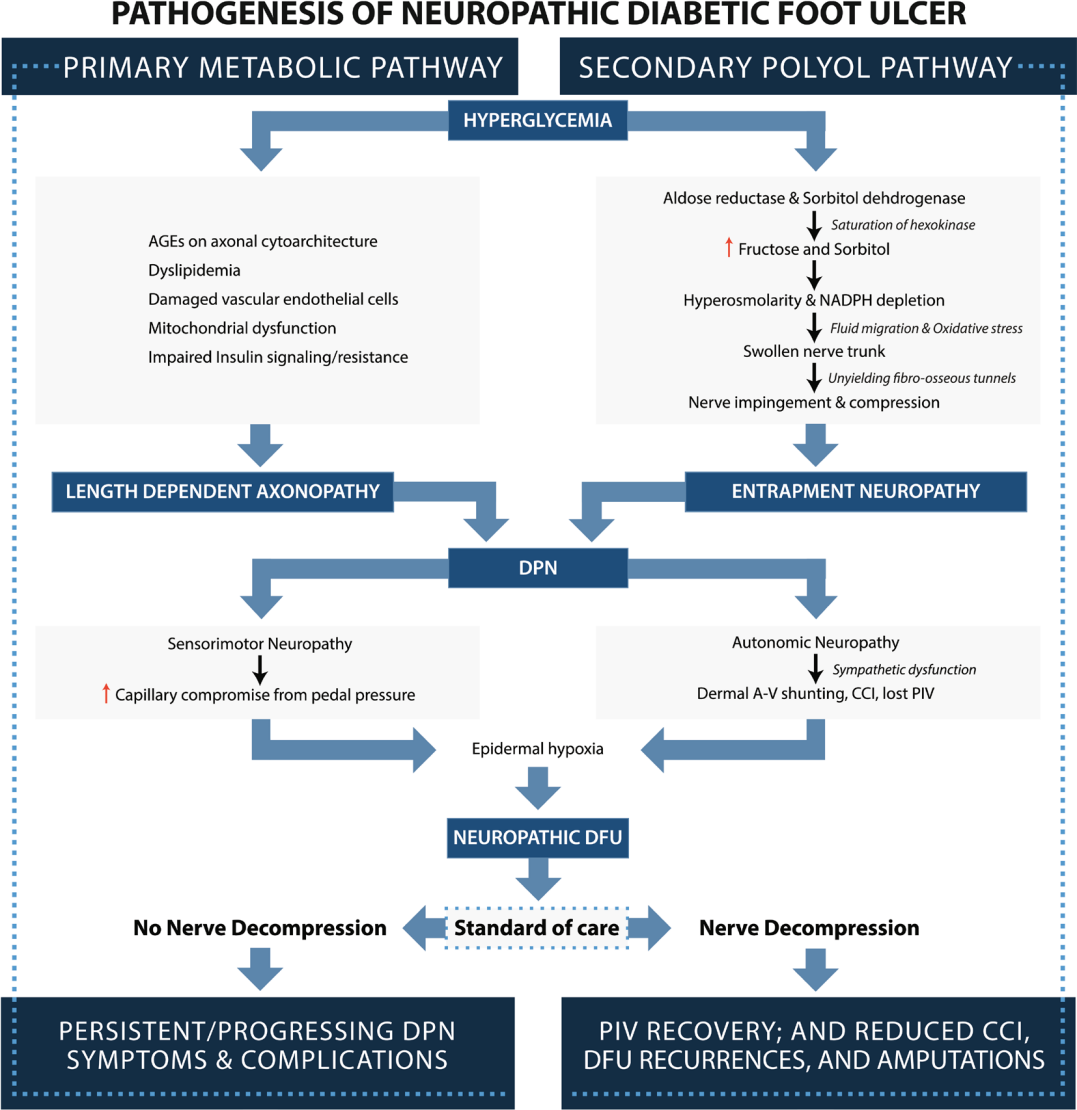
DPN is often described as a length dependent axonopathy resulting from a dysfunctional metabolic pathway shown on the left. The polyol pathway, shown on the right, is also well‐known and results in an increased concentration of fructose and sorbitol within the nerve and myelin. This results in hyperosmotic swelling of the nerve and entrapment neuropathy (EN) at several fibro‐osseous tunnels in the lower extremities. EN can explain both the sensorimotor and autonomic deficits seen with DPN including dermal a‐v shunting, CCI, and diminished PIV, which can all be improved with nerve decompression.

Our schema in Figure 4 offers a more complete depiction of DPN. It illustrates the universally recognised LDA metabolic pathway and acknowledges the contribution of the secondary polyol pathway which causes nerve swelling, local compressions, EN and dysfunction of peripheral nerves in DPN.

**FIGURE 5:**
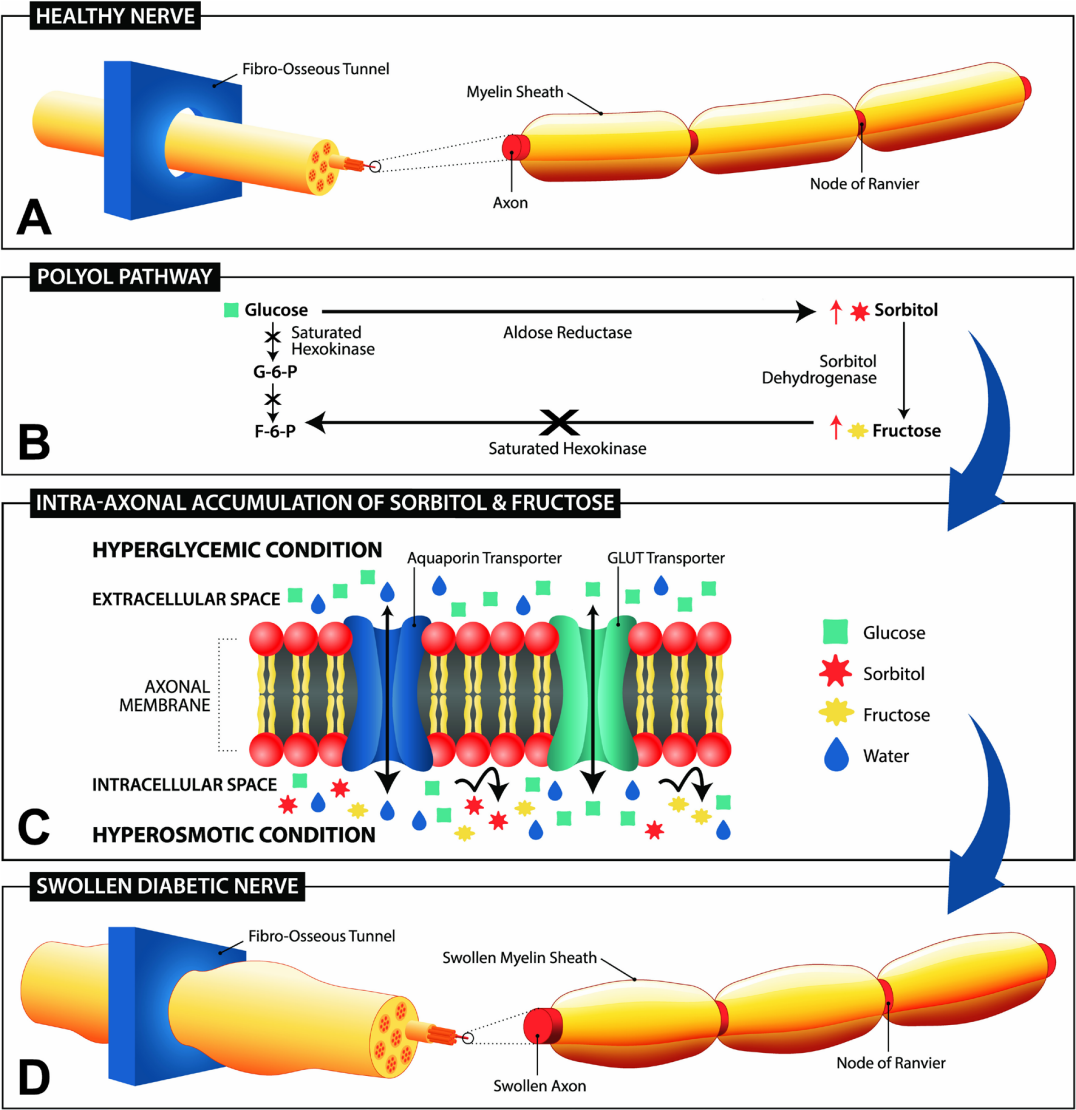
(A) In the healthy nerve water and glucose equilibrate across the axonal membrane bidirectionally by diffusion and/or transport. (B) In diabetes, hyperglycaemia drives the polyol metabolic path to accumulate sorbitol and fructose. (C) Sorbitol and fructose have no membrane transporters and accumulate inside the axon. Water or tissue fluid is drawn across the membrane into the axons to equalise osmolarity. (D) This causes a swelling of the myelin sheath, axons, fascicles and nerve diameters. The healthy nerve glides easily through the fibro‐osseous tunnel (A) while the swollen nerve suffers measurable compression (D).

Figure 5 is an artistic rendition of how hyperglycemia and the polyol metabolic pathway result in a hyperosmotic condition with swelling of the nerve. The result is an entrapment at the fibro‐osseous tunnels through which the nerves pass. This explains how ND surgery can be so effective in treating DPN patients. EN in diabetes generates both somatic sensorimotor loss and a distal autonomic neuropathy which produces the epidermal microcirculation deficits [40, 41]. Recognition of EN's presence in DPN is crucial and consequential because focal nerve compression and the associated autonomic dysfunction are responsive to surgical nerve decompression (ND) [18, 40, 42]. A more complete physiologic perspective on DPN and the causes of DFU should acknowledge the literature connecting the cutaneous hypoxia to EN with swelling of the myelin sheath [43], axons, fascicles and nerve diameters and subsequent autonomic effects on microcirculation.

### 3.2 Connecting DPN, Microcirculation, Foot Complications and ND Surgery

Dellon had suggested using ND for relief of painful DPN before 1990 [8]. First evidence that external neurolysis for ND might protect against DFU and amputation events was presented by Azsmann et al. in 2004 [44]. Their unexpected finding was that, in a painful DPN cohort, every DFU or amputation which followed a unilateral ND surgery for pain relief appeared in the non‐operated contralateral limb. Thirty per cent of the non‐operated legs developed a first DFU or toe amputation in the mean 4.5 years of F/U. Operated ND legs had none. The physiologic basis of this apparent protection was unexplained. Return of protective skin sensibility was hypothesised to be implicated through encouraging more position shifting and plantar unweighting [45, 46, 47]. Neuro‐circulatory effects were not at first suspected of involvement.

Many historical reviews of neurovascular deficits associated with diabetes and DFU have discussed a potential microcirculatory connection to DFU risk [7, 14, 16, 17, 48, 49, 50, 51]. While the deficit of skin microcirculation in diabetes with DPN and DFU has been recognised for four decades, no therapeutic interventions have been proposed to correct the situation [51]. Now, however, ND has been found to measurably improve epidermal microcirculation in diabetes with EN, in both clinical and laboratory situations [7, 18, 22, 24, 40]. Recognising the frequency of EN in DPN and the therapeutic effects of ND, we can begin to unravel how this surgery offers great potential for neurovascular benefits.

### 3.3 ND And Risk of Initial DFU or Recurrence

Medical literature advises an expectation of 22%–40% DFU recurrence within a year of successful SOC therapy [34, 52]. The 19 papers of a NEJM meta‐analysis describe new wounds occurring in 40% of the 3329 IWGDF Risk Group 3, prior DFU subjects at 1 year and in nearly 60% by 3 years [34]. A second global meta‐analysis reported 22% annual recurrence [52]. But ND studies show an unexpected, much lower nDFU recurrence rate. Table 1 lists seven prospective and retrospective observational studies finding that incidence of recurrent nDFU is merely 1.39% in 573 Risk Group 3 cases that have undergone lower extremity ND [58]. Included studies have had bilateral or only unilateral ND. No amputations occurred in the treated legs in 1–3‐year F/U.

**TABLE 1:** nDFU recurrence and amputations in ND studies.

Study authors	ND Cohort (IWGDF Class 3)	Active nDFU at ND	Healed during F/U	Recurrent DFU	Study follow‐up (years)	Patient follow‐up years	Annual RECURRENCE Risk	Amputations post‐healing
Dellon et al. 2012 [53]	57	0	^b^	2	1	57	3.51%	0
Nickerson 2010 [54]	75	4	4	8	2.5	187.5	4.27%	0
Nickerson and Rader 2014 [55]^a^	42	0	^b^	2	3	126	1.59%	0
Trignano et al., 2015 [42]^a^	8	8	8	0	1.5	12	0.00%	0
Viswanathan et al., 2018 [56]	61	61	61	1	1.5	91.5	1.09%	0
Zhang et al., 2013 [46]^a^	206	0	^b^	0	1.5	309	0.00%	0
Liao et al., 2014 [57]^a^	77	0	^b^	0	2	154	0.00%	0
Total	526 (859 legs)	73	73 (100%)	13	NA	937	1.39% 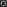	0

*Note:* Seven studies report occasions of DFU recurrence after ND surgery in a healed ulcer IWGDF Risk Group 3 cohort. 73 cases with open ulcers at surgery all healed after ND. After initial healing, only 13 ipsilateral new DFU appeared in 937 patient‐years of F/U. Duration of F/U ranged from 1 to 3 years (mean 1.78 year). There were no amputations in any study. 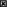
Annual recurrence risk = 1.39%.

^a^ Represents bilateral ND surgery.

^b^ Represents ulcers that had healed before surgery.

### 3.4 ND And Neuro‐Ischaemia in DPN

A clinical study suggests ND surgery can also be useful for improving microcirculation in diabetic neuro‐ischaemia. Trignano et al. reported on 40 ft of 20 patients with mild PAD and clinically abnormal pre‐operative tcpO2 of < 40 mmHg [42]. The cohort of 20 included 8 cases with an open DFU at the time of surgery. Bilateral ND improved the CCI hypoxia in every leg and produced a final tcpO2 of ≥ 40 mmHg in 38 of 40 legs (95%) at 18 months. Every neuro‐ischaemic DFU (n‐iDFU) wound healed, and no new DFU, amputation or revascularisation occurred in any leg. TcpO2 recovery implies ND surgery should benefit the skin oxygenation deficit adjacent to a n‐iDFU, which Nouvong et al. have demonstrated using hyperspectral oxyHgb imaging to be strongly correlated with DFU healing [59]. Clinical recovery of measured PIV after ND in DPN or nDFU has not yet been evaluated in humans, but it does occur in rats [16].

### 3.5 ND Effects in Animal Models of EN

Deficits of both CCI and PIV microcirculation develop in a rat diabetes laboratory model which creates sciatic EN that can later be released [7, 18, 22, 40, 50, 60, 61, 62]. This model evaluates several objective outcomes measured after periods of sciatic compression. These include NCV, cold temperature sensitivity, sensory latency for paw withdrawal, histologic axonal degeneration and demyelination and the degraded epidermal capillary blood flow of CCI and PIV. Subsequent ND resulted in at least partial restoration of each abnormal parameter [7, 18, 22, 40].

### 3.6 SOC Therapy for DFU; Focus on Microvascular Issues

Recognising the complexity of DFU aetiology, we must appreciate that the SOC for prevention, active DFU treatments or healed ulcer aftercare chiefly targets patient self‐surveillance, incident skin pressure, concurrent infection, the local wound bed environment and macrovascular PAD problems. Short leg walking casts, custom footwear and orthotic inserts can all modify local plantar pressure elevation and concentrations as well as three‐dimensional skin shear stress. Footwear changes do allow most non‐plantar ulcers to heal [3]. Some wound dressings can improve healing rates [63, 64]. Arterial stenoses with severe PAD often respond to surgical revascularisation, though such procedures provide only incomplete restoration of cutaneous microcirculation [65]. It is encouraging that historical restenosis rates of up to 55% in diabetes cases are said to have decreased to < 20% as drug eluting stents and balloons have replaced atherectomy, balloon angioplasty and bare metal stents [66]. However, the extent of DPN and the microcirculation insufficiency are unchanged by any of these SOC interventions. Persisting EN, sympathetic dysfunction, CCI, and PIV loss can be implicated in the common failures to heal, desultory DFU wound closures and high recurrence risk.

### 3.7 DFU Recurrence Site Location

A subsequent DFU can occur at the previous site, a new ipsilateral location or the contralateral foot. The site of recurrence might be expected to be most frequently at the original location, where the aggregate causal factors first produced skin failure. Yet the next ulcer after DFU healing is most often at a new site. The two studies by Orneholm et al. and Peterson et al. report that after good SOC treatment methods, Risk Group 3 recurrence location is at a same or new ipsilateral site in around 55%–60%. Identical site recurrence was < 20%. Their combined experience observed 161 of 391 subsequent ulcers (41%) were located on the contralateral foot [67, 68]. Engberg et al. found total recurrent and new ulcer risk to be somewhat less frequent in nDFU vs. n‐iDFU wounds (30.6 vs. 36.7 per 100 person‐years) [69]. The two ND studies that compared new ipsilateral vs. contralateral nDFU recurrence sites show that post‐surgery, the operated leg experiences a lower risk of the next DFU. After unilateral ND with 2–5 years F/U, nDFU recurrences appeared in 12% (9/75) of operated limbs and in 30% (16/53) of non‐operated contralateral legs [56, 70]. So, contralateral recurrence was 2.5 times more common. Other investigations with 12–24 month F/U of 293 Risk Group 3 cases with bilateral surgery found no subsequent ulcers occurred on either foot [42, 46, 57]. Such results imply that bilateral ND would be required for the most effective minimising of risk for any new DFU in IWGDF Class 3 patients. A Level 1 study is needed to further test this.

### 3.8 Amputation After DFU

DFU is an independent risk factor for both amputation and mortality [71]. In IWGDF Risk Group 3 subjects treated with ND after wound healing, the operated leg has strong protection from amputation. Moulik et al. report 5‐year amputation risk after SOC for nDFU without ischaemia to be 9.6% (8/83) [72]. None of the 7 ND studies in Table 1 reported even one amputation of an operated nDFU leg during shorter term F/U of 1–2 years. The single cohort of 65 Group 3 subjects followed longer, for 5 years, found no amputation had occurred in the 75 operated legs [70].

### 3.9 Hyperbaric O2 Treatments and Hard‐To‐Heal DFU

Hyperbaric oxygenation treatments for DFU have been advocated to address the recognised skin hypoxia related to recalcitrant healing of the DFU. Benefits have been inconsistent. Frykberg et al. presented a prospective RCT attempting to definitively settle this question in desultory healing wounds [73]. Yet that study, too, was subject to criticism as inadequate [74]. It is unsurprising that topically applied or pressurised external oxygen might yield limited effect when impaired epidermal microcirculation persists due to the ongoing sympathetic damage, a‐v shunting, curtailed capillary microcirculation and reduced PIV.

A minority of patients in DFU SOC reports have a prolonged, desultory healing course or remain unhealed until patient demise. Recalcitrant, ‘hard‐to‐heal’ wounds are defined as showing < 50% reduction in wound surface area over 4 weeks of SOC [64]. Orneholm reports 21% of 701 DFU with SOC treatment require amputations or fail to heal before death [75]. Gershater found 35% having the same results [76]. A larger RCT reports that 34% (45/132) of ‘hard‐to‐heal’ wounds closed in 20 weeks with SOC plus autologous platelets, fibrin and leucocytes, using the *3C Patch* (formerly LeucoPatch). They realised only 22% success (29 of 134) with SOC alone after 20 weeks F/U. Armstrong et al., selecting a RCT cohort having < 30% size reduction in only 2 weeks of SOC, report healing 70% in 12 weeks with an autologous skin construct vs. 34% who healed in his continuing SOC control group [77]. None of these studies provide any data on recurrence rate or patient survival in longer‐term F/U. However, a study from France has found that duration of ulcer is a significant predictor of mortality, and patients with long‐term non‐healed ulcers are at high risk of death within 5 years, independent of PAD or age [78]. More promisingly, Pejkova et al. presented at the EURAPS 2023 conference a finding that 67% of long‐term ulcers—unhealed for a median 9 months—had closed within 7 months after tarsal tunnel ND (personal communication). The remaining 1/3 have reduced wound area, and posterior tibial artery flow measured 60% higher. ND may offer value in realising closure of these ‘hard‐to‐heal’ situations by restoring microcirculation.

## 4 Discussion

The most interesting result from this review is that nDFU annual recurrence risk after ND is < 2%. Two meta‐analyses of SOC results report 20%–40% risk [34, 52]. A 90% reduction in DFU recurrence would have major personal, medical and financial consequences. Wider understanding of the convoluted and complex path from diabetes to foot complications is necessary to realise the potential of ND.

### 4.1 Objective ND Benefits

The C‐fibre and A‐delta neurons are considered to be principal regulators of neurovascular functions [25, 79, 80]. There is laboratory and clinical evidence that ND improves CCI, electrophysiology [22, 57, 81, 82], and restores PIV [18, 19, 20]. This could have its basis in peripheral nerve regeneration or alternatively, an unexpected immediate neural functional recovery. Three clinical studies of DPN complications found a significant improvement of NCV deficit at 18 months after ND, while non‐operated control patients continued with deficiencies [46, 57, 83]. It is known that peripheral nerve recovery from neurotmesis proceeds at a rate around 1 mm/day. However, relief of local compression has been shown to unexpectedly allow *immediate* improvement of EMG and neural circulation function. Anderson et al. demonstrated in 82.6% of operated DPN legs an immediate (within 2 min after ND) and significant mean improvement of 73.6% in intraoperative EMG values (*p* < 0.0001). This occurred within the brief 60–90 seconds required to measure motor evoked potentials after completing common peroneal nerve release in diabetic subjects [81]. Chronic EN with compression in diabetic mice decreases vasa nervorum flow but both flow and NCV improve with early ND [24]. Concomitantly in humans, Uemura et al. measured > 30% immediate increase in SPY‐measured indocyanine green fluorescence of perineural blood flow after the brief, minutes‐long duration of endoscopic tibial nerve ND at the tarsal tunnel [23]. How these extremely rapid improvements in objective EMG and vasa nervorum flow occur is not yet understood. Whether the same immediate response is true for NCV and skin CCI and PIV has not been studied clinically.

In further support of ND importance, rat studies show that progressive medium‐term histological improvement in arterial micro‐vessels and nerve myelinisation complement the ongoing and progressive NCV recovery after removal of sciatic compression [22, 40]. Demiot et al. found that chemical suppression of the aldose reductase metabolic pathway prevents loss of PIV in mice [79]. This suggests that the intraneural accumulation of sorbitol and the nerve enlargement which results in EN has been reduced or prevented chemically. Unfortunately, aldose reductase inhibitors have been disappointing for producing recovery of PIV in clinical trials [51, 84].

### 4.2 Further ND Opportunities

Potential clinical applications of ND in DPN are manifold. Current SOC therapy and recommended aftercare for healed DFU do not address ongoing EN compressions, the sensorimotor deficits or the autonomic skin microcirculation defects. Certainly, there is proven benefit from the plantar pressure offloading, which is essential in SOC treatments [85], despite the baseline and continuing presence of DPN‐based CCI and PIV loss. That improvement presumably derives from a local reduction of external, mechanical capillary compression and any flow restriction which occurs with ambulation and standing. Improvement of autonomic function, CCI and PIV after ND would be additive to this proven importance of SOC plantar pressure unloading.

ND could be considered for prophylaxis of DPN cases at risk of either initial or recurrent DFU, using bilateral surgery to maintain PIV and prevent CCI, skin breakdown and loss of protective sensation [42, 46, 57]. Such avoidance of initial ulceration is suggested by Azsmann et al. [44], Zhang et al. [46], and Dellon, Muse et al. [53] Prophylactic screening for potential to ulcerate is likely possible as well. A rapid, non‐invasive, hyperspectral oxy/deoxy Hgb skin imaging technique is available which can identify an area of incipient ulceration 2 months in advance of wound appearance [86]. Plantar temperature monitoring (PTM) is said to provide > 1 month advance prediction of DFU appearance [87]. Routine PTM or hyperspectral scanning of both feet for all IWGDF Risk Class 2 and 3 diabetic patients could identify new ipsilateral or contralateral sites potentially at risk before actual skin breakdown and DFU wound development occur. This would allow an opportunity for prophylactic ND, in legs diagnosed as having EN, before a first DFU or recurrence appears. The large expenses of treating infected wounds, sluggish DFU healing and the associated late complications could then be avoided.

ND could bring added value to initial SOC treatment for nDFU, now consisting largely of wound debridement and environment optimising, pressure unloading, activity constraint and infection management. Performing ipsilateral ND alone would fail to reduce the significant 40% risk of contralateral appearance of a next ulceration [67, 68, 76]. Gershater et al. found that primary wound closure without any amputation was accomplished in 78.4% of 2060 healed DFU, with 49% being neuro‐ischemic [76]. While this result is consistent with other SOC studies, Viswanathan et al. showed that ND plus SOC healed 90% of first nDFU wound cases (*n* = 61) in 12 weeks and 100% within 6 months, with no amputation or mortality at 1.5‐year follow‐up [56]. That 90% healing rate in 12 weeks supports the notion that adding ND to SOC might accomplish more rapid and frequent healing for the nDFU wounds.

If larger RCT studies confirm the encouraging success of Trignano et al., ND will reduce the risk of new n‐iDFU in DPN [42]. In their study, they demonstrated that (1) ND significantly improves microcirculation using objective tcpO2 measurements, (2) all pre‐operative DFUs healed and (3) no recurrent DFU occurred at 18 months of follow‐up. Correlation of ulcer healing to local skin oxygenation has been demonstrated [59] and helps explain the Trignano et al. result. Recovery of microcirculation might improve on the 5‐month SOC healing rate of 34% that Game et al. report, which is the best result reported and published for the recalcitrant, indolent, hard‐to‐heal nDFU [64]. Pejkova's small study finds 67% of such recalcitrant nDFU cases healed within 6–9 months after tarsal tunnel ND. She also reports posterior tibial artery flow increased by > 60% (personal communication). Tekin similarly found a 17% arterial flow increase [21]. The *p*‐values for both studies were ≤ 0.05. Tekin stated improved pulsatility and lower flow resistance occur as well. It appears that routine inclusion of ND with revascularisation for PAD in n‐iDFU should enhance both distal arterial runoff and microcirculation benefit as Blount et al. suggest [88]. This could hardly be detrimental and raises the question of whether ND should be routinely employed in diabetic neuro‐ischaemia situations.

If effective enough, better nourishment and oxygenation of skin could minimise necessity of some SOC measures and expenses like custom footwear, pressure modifying insoles, prolonged PTM and adherence to activity reductions. Future research may find less frequent DFU aftercare exams to be necessary when more normal microcirculation is restored by ND. For Charcot neuroarthropathy, two studies have suggested that ND in early Eichenholtz stages 0 or 1 can avoid progression to the later stages having loss of foot architecture [83, 89]. The frequent ulcer wounds and recurrences associated with Charcot foot skeletal deformities might be minimised thanks to osteoclasis suppression or microcirculation revival with better skin nutrition and oxygenation post‐ND. More resilient epidermis could improve outcomes with cheilectomy and avoid necessity for some major reconstructions and amputations. This topic begs for future research attention.

### 4.3 ND And Surgical Site Infection (SSI)

SSI has been a cautionary criticism of those sceptical of using ND for painful DPN. The concern of the Cornblath et al. critique [90] about high risk of surgical site infection in DPN patients with the ND surgical incisions has not been observed. Around 10% risk of surgical site infection (SSI) is presumed with foot and ankle surgery for diabetic patients [91]. Studies showing that surgical site infection in DPN relates more to neuropathy than the diabetes diagnosis ease that concern [92]. Incisions at multiple entrapment sites have not caused significant SSI risk with ND [46, 53, 57]. The explanation for this result is a topic for further research. Lack of SSI combined with improved microcirculation suggests the current opinion that the healed DFU is only ‘in remission’ [93, 94], may be too pessimistic.

### 4.4 Plantar Temperature Monitoring

Plantar temperature monitoring (PTM) after DFU healing is currently of interest. It has lowered recurrence risk around 50% in some research situations and claims the ‘highest reported reduction in incidence of foot ulcer recurrence compared to usual care of all preventive strategies’ [95]. The PTM strategy identifies skin temperature elevation associated with activity and is thought to provide early warning of local inflammation and incipient skin failure. The appearance of a ‘hot spot’ allows counsel for weight‐bearing restrictions until local skin temperature elevation has resolved. However, patient compliance with activity restrictions can be low. A study of unsupervised use of PTM in a cohort of 151 Risk Group 3 cases found that only 1/3 of the patients were following advice to reduce activity by 50% when a hotspot is found. Annual DFU recurrence risk in these conditions was a disappointing 20% [96]. Another study was similarly discouraging, finding that 29 of a 129 case Risk Group 3 cohort (22.5%) experienced 53 new ulcers in only 34 weeks of unsupervised PTM [87]. Effective use of PTM apparently requires ongoing support infrastructure, long‐term use, and persistent adherence to activity restrictions. No lasting protection is to be expected without continued adherence [97, 98, 99]. Aan de Stegge et al. now question the association of incipient DFU and skin temperature, finding that more than half of new ulcerations are not immediately preceded by local skin temperature elevation [100]. In contrast to PTM, ND has the potential advantages of being immediately therapeutic to CCI and lost PIV and durable without further management required. Thus, after ND, future costs of investment in infrastructure and DFU aftercare should be reduced [101] and QOL improved [102].

Reviewing an expanded DFU aetiology in DPN, we can begin to hypothesise the relative importance of several causative factors. Surely impaired pedal light touch hinders self‐recognition of plantar tissue being damaged and allows overuse. ND improves sensory light touch, 2‐pt discrimination, and vibration perception, which facilitates tactile feedback for DFU prevention and intact skin maintenance and should be helpful [46]. It has been demonstrated that merely walking for several minutes will raise pedal skin temperature to 35° centigrade. Such temperatures increase epidermal nutritional demand, encourage tissue breakdown and discourage cutaneous repair [103, 104]. Improved capillary blood flow to the epidermis following ND would minimise and mitigate those activity‐related skin temperature increases. Improving the foot sensibility, CCI, epidermal nutrition, skin hypoxia and PIV through the use of ND would combine neurovascular benefit with established SOC to produce better overall DFU treatment outcomes.

### 4.5 Recommended Interventions for DFU

Van Netten et al. reviewed 72 publications (26 with controlled and 46 non‐controlled study design), which evaluated ‘modifiable risk factors for treatment’ of DFU [105]. They conclude that: ‘structured education for patients and health care professionals, callus removal, custom‐made therapeutic footwear, and foot‐ and mobility‐related exercises may be beneficial for improving modifiable risk factors for foot ulceration. However, we generally found low quality of evidence for interventions targeting modifiable risk’. Microcirculation enhancement with ND was not one of the suggested treatments for modifiable risk factors. Prospective controlled studies need to confirm the strong neurovascular benefits observed in laboratory and clinical observation reports of ND use. Furthermore, evidence of persistent new ulcer risk in an unoperated leg suggests bilateral surgery is required for holistic protection. ND surgery may be an important addition to SOC treatment through its ability to amend compromised skin circulation and thus minimise DPN, prevent DFU and improve the bleak outlook for serious diabetic foot complications.

### 4.6 Strengths and Weaknesses of the ND Literature

#### 4.6.1 Weaknesses

DFU research in toto suffers from inconsistency in the classification of wound types and patient factors like neuropathy and neuro‐ischaemia. Past studies may have used Meggitt‐Wagner, PEDIS, SINBAD, SEWSS, University of Texas or WIfI classifications. Almost all ND ulcer studies involve only neuropathic nDFU and not the frequent n‐iDFU wounds. This limitation respects the patient selection criteria for ND, which advise that surgical candidates should have adequate circulation with palpable pulses or ABI value > 0.7 [8]. Yet the worldwide DFU spectrum includes 1/3 to 2/3 having variable degrees of ischaemic macrocirculation [106]. Only the ND cohort of Trignano includes n‐iDFU or impaired tcpO2 cases. Yet those results were also impressive for tcpO2 improvement, DFU healing and avoiding recurrence or amputations. There are no ND studies that address the widely appreciated problem of kidney disease and‐associated DFU, so improving microcirculation in these cases is currently of unknown potential [2].

The predominant critique of the ND surgery for DPN is the lack of Level 1 EBM studies. Recently, Rozen et al. have published the first Level 1 study of ND [107]. The primary endpoints were *subjective* QOL and relief of pain, which were both significantly improved. Most reports of ND in DFU wounds are Level 2A or 2B prospective or retrospective observational reports. Viswanathan et al. is a prospective randomised protocol but lacks a SOC control group. Critics' continued hesitance about surgery in DPN has been directed at use of ND to address *subjective* pain outcomes. The sceptical appraisal still holds sway today in judging the quantitative, *objective* DFU outcomes we are reviewing. The data in Table 1 all refer to *objectively* measured outcomes, making the bias, placebo and diabetes natural history critiques of Cornblath rather less compelling [90]. Our new understanding of ND and autonomic neural recovery explains improved sensorimotor and neurovascular function, including epidermal capillary and larger artery flow.

Furthermore, the ND ideas were coming from an academic domain, i.e. plastic surgery, having little past involvement with the metabolic hypotheses and academic concepts about diabetes and DPN aetiology and complications. Contrarily, primary care diabetes physicians had little personal experience with the swollen, unhealthy appearance and local compressions of diabetic nerves, which are so impressive to surgeons in the operating theatre. Diabetologists have recommended that ND should not be considered until prospective, randomised controlled trials are provided that included sham surgery to minimise placebo effects. The Rozen et al. study now delivers EBM Level 1 evidence for relief of DPN pain [107], and this review presents the Level 2 evidence for DFU recurrence and amputations.

#### 4.6.2 Strengths

The strength of this review is that it provides objective evidence of beneficial ND effects. It proposes a more complete description of aetiology and the pathophysiologic processes involved in progression from diabetes to DPN and the diabetic foot complications. That link to DFU complications is why ND surgery is important. It unveils the physiology‐based scientific rationale for using surgery to rejuvenate skin and nerve microcirculation. The ND reports have found uniform resolution or reduction of DPN complications via the surgical elimination of EN. The polyol metabolic pathway is not eliminated, but its neurologic effects are minimised. Each step in this process is described and objectively demonstrated in the scientific literature referenced. The critique of Ioannidis that 80% of medical research cannot be replicated [108] becomes less powerful as the numbers of studies supporting the use of ND increase and the putative negative results or surgical complications fail to appear.

Widespread use of ND could have immense patient and economic benefits worldwide. With an estimated 1/3 of the USA's $237 billion expense of diabetes (2017) being due to lower extremity and foot care problems, ND would seem to offer great potential for cost savings [94]. One study estimated several billion dollars in potential savings for the US alone if ND were routinely employed for DFU [101].

No other effective treatment of deficient microcirculation currently exists [51]. Regardless of physiological uncertainty about precise details of DPN aetiology, the clinical and laboratory evidence documenting the benefits of peripheral nerve surgery in the diabetic foot seems very encouraging. The ND intervention is directed at an accepted neurovascular pathology and improves important microcirculation manifestations of DPN. Clinical observations show large ND effects on the incidence of initial DFU and nDFU recurrence risk, amputation risk and post‐healing mortality. Verification of objective improvement in microcirculation, electrophysiology and laboratory histological measures is established. Deficits of motor evoked potential, NCV, CCI, PIV, perineural circulation and foot sensibility are all improved. ND directly improves those nerve compression symptoms and consequences [38].

It is interesting to contemplate why there has been relatively minimal interest in applying the surgical ND tactic to DPN and its DFU complications. Certainly, the presence and significance of the EN in diabetes has been minimally recognised or considered by non‐surgical academia. The current ADA Professional Practice Committee's scientific analysis of advanced wound therapies does not mention ND among its list of more than 30 options in 11 broad categories [109]; nor does another current article addressing psychosocial care for the pain and DFU complications of DPN [110]. ND studies are rarely reported in journals targeting wound care, neurology, endocrinology or primary care practitioners. Those aware of the low recurrence risk reports may be sceptical of them without Level 1 studies and some connection to a credible aetiology [90]. There is no medical device or pharmaceutical market associated with the use of ND surgery which would attract industry financial support. Currently, only surgeons and their patients stand to benefit. With ND in the toolbox, all DPN‐related specialists can evaluate outcomes, and many more patients can potentially benefit.

The link of a‐v shunting and epidermal microcirculation impairment has long been known. Only now is the role in DFU of the secondary polyol metabolic pathway, peripheral nerve swelling, fibro‐osseous tunnel impingement and compression neuropathy being emphasised [58, 107]. With recognition of the beneficial skin microcirculation effects associated with ND, there is potential for this oversight to change. This compilation of published laboratory and clinical evidence connecting ND, minimised DFU recurrence risk, and low subsequent amputation rate hopes to encourage such an evolution.

## 5 Conclusion

Hyperglycaemia in diabetes augments a secondary polyol metabolic pathway that produces enlargement of peripheral nerves. The swollen nerves become compressed in the unyielding fibro‐osseous tunnels through which they glide. This nerve compression affects both large myelinated and the small A‐delta and c‐fibres that participate in reduced sensation, motor weakness, pain perception and neurovascular control of skin microcirculation. All of these compressed fibres benefit from ND. One result is a robust effect from surgical ND in reducing nDFU recurrence in multiple studies to 1.39% annually.

The relentless worldwide progression of incident diabetes, DPN and the cascade of associated diabetic foot wounds, amputation and early mortality complications is discouraging. Only ND has shown any improvement of the recognised microvascular dysfunction causing epidermal ischaemia. Rejuvenation of skin microcirculation, in combination with sensory improvement, seems an attractive pathology‐based mechanism of action for ND. The worldwide predicament of diabetic foot complications may be noticeably curtailed by adding low‐risk, outpatient ND epineurolysis surgery to the diabetologist's current SOC tools for addressing DPN and diabetic foot complications.

## Ethics Statement

We have reviewed the ‘Guidelines on Publishing and Research Ethics in Journal Articles’ and find there are no conflicts.

## Conflicts of Interest

Dr. Nickerson has no conflicts to declare. Dr. Yamasaki is a co‐founder and president of Enso Medical Technologies Inc., which did not contribute to or fund any part of this review. Thus, there are no conflicts for either author.

## Permission to Reproduce Material from Other Sources

Dr. Ryan Periera has given written permission to reproduce the photo in Figure 3. Prof Gun Jorneskog has given written permission to reproduce the CCI illustrations in Figure 1.

## Data Availability

The data that support the findings of this study are available from the corresponding author upon reasonable request.
